# Biological Properties, Phenolic Profile, and Botanical Aspect of *Nigella sativa* L. and *Nigella damascena* L. Seeds: A Comparative Study

**DOI:** 10.3390/molecules28020571

**Published:** 2023-01-06

**Authors:** Leila Benazzouz-Smail, Sabiha Achat, Fatiha Brahmi, Mostapha Bachir-Bey, Radia Arab, José Manuel Lorenzo, Aicha Benbouriche, Kahina Boudiab, Didier Hauchard, Lila Boulekbache, Khodir Madani

**Affiliations:** 1 Laboratoire de Biomathématiques, Biochimie, Biophysique et Scientométrie, Faculté des Sciences de la Nature et de la Vie, Université de Bejaia, Bejaia 06000, Algeria; 2 Laboratoire de Biochimie Appliquée, Faculté des Sciences de la Nature et de la Vie, Université de Bejaia, Bejaia 06000, Algeria; 3 Centro Tecnológico de la Carne de Galicia, Rúa Galicia Nº 4, Parque Tecnológico de Galicia, San Cibraodas Viñas, 32900 Ourense, Spain; 4 Área de Tecnología de los Alimentos, Facultad de Ciencias de Ourense, Universidad de Vigo, 32004 Ourense, Spain; 5 Ecole Nationale Supérieure de Chimie de Rennes, CNRS UMR 6226, 13 Allée de Beaulieu, CS 50837, CEDEX 7, 35708 Rennes, France; 6 Centre de Recherche en Technologies Agro-Alimentaires, Route de Targa Ouzemour, Bejaia 06000, Algeria

**Keywords:** *Nigella sativa* L., *Nigella damascena* L., seeds, botanical study, chemical composition, biological activity

## Abstract

The use of *Nigella* seeds in the food, pharmaceutical, and cosmetic fields is common, since the iniquity and the virtues of these plants are directly related to their characteristic phytochemical composition. This investigation focused on the comparative study of the botanical aspect, phenolic profile, and in vitro and in vivo biological activities of *Nigella sativa* L. (NS) and *Nigella damascena* L. (ND) seeds. The macro- and micro-morphological properties of these seeds were studied, and the key dissimilarities between them were clearly illustrated. The phytochemical contents and phenolic profiles were determined, and the in vitro antioxidant activity was assessed using four methods. The in vivo antioxidant and biochemical parameters of the blood of supplemented mice were determined. The results of the macro- and micro-structure analysis revealed differences between the two plants. Here, ND is characterized by higher phytochemical contents and the best antioxidant activities. The HPLC analysis indicated the presence of nine compounds, namely seven phenolic acids, particularly hydroxybenzoic and caffeic acids, and two flavonoids. The administration of ND extract to mice for 21 days at a concentration of 500 mg/kg allowed a substantial amelioration of plasma antioxidant properties. In addition, the extracts ameliorate blood parameters (cholesterol, triglycerides, glycemia, and urea). Furthermore, the antimicrobial activity of extracts demonstrated their effects on *Staphylococcus* and *Aspergillus*. *Nigella* seeds, in particular ND, expressed considerable in vitro antioxidant properties and demonstrated significant amelioration of mice blood properties. Consequently, these species can serve as a valuable source of compounds with various applications.

## 1. Introduction

The genus *Nigella* includes about 22 species of annual herbs belonging to the buttercup family (Ranunculaceae) [1]. The term *Nigella* comes from the Latin “*niger*”, a reference to the intense black color of seeds in most *Nigella* species; the best known of these species are *Nigella sativa* L. (NS) and *Nigella damascena* L. (ND) [2,3,4]. It is well known that both species are widely used in the Mediterranean and in Asian traditional medicine, as well as in industrial applications and for their culinary properties.

*Nigella sativa* L. is commonly known as black cumin, black seed, habba Saouda and senoudj or sinoudj in Algeria and the Maghreb countries. It is naturally grown in North Africa, southern Europe, the Middle East, and Southwest Asia, and is widely cultivated for numerous industrial and medicinal purposes (food and therapeutic uses) [1,5,6,7] mainly in India, Pakistan, Iran, Syria, Turkey, Ethiopia, Oman, and Saudi Arabia [8,9,10]. The NS seeds stimulate great interest in the food, cosmetics, and pharmaceutical fields. They are applied for respiratory and digestive disorders including asthma, bronchitis, dysentery, and stomachache, and are is utilized as an insect repellent, diuretics, and for the treatment of rheumatism, headaches, as well to improve kidney and liver function [1,2,6,11]. The NS seeds are also employed as preservatives and spices to flavor foods, such as bakery products, cheese, pickles, and a variety of traditional dishes [4,8,12].

Recent scientific researches indicate several bioactivities for NS seeds, including antimicrobial [13], anti-histaminic [14], anticancer [15,16], anticonvulsant [17], anti-inflammatory [2,18], anti-diabetic [19], antihypertensive [7], diuretic, and antioxidant activities [20]. The mentioned biological properties are related to their richness in several phytochemicals, mainly phenolic compounds, terpenes, essential oils, saponins, and alkaloids [7,20,21]. The NS seeds are also a substantial source of nutritionally vital constituents, such as fixed oils (32.2–41%), as well as to the fact that seeds are rich in polyunsaturated fatty acids, proteins (13.8–22%), carbohydrates (17%), ashes (4.5–7.5%), dietary fibers (8–16.4%), and vitamins, such as tocopherol and niacin [7,22,23,24,25,26].

*Nigella damascena* L. is commonly known as love-in-a-mist, or nigelle de damas (in French). It originated from southern Europe and is widespread throughout temperate regions of Europe and the Mediterranean region, and it is cultivated worldwide, especially as an ornamental plant [2,6,27,28]. The ND seeds are used in traditional medicine because of their analgesic, diuretic, antipyretic, vermifuge, and disinfectant effects and to regulate menstruation and catarrhal affections [6,20,29,30]. They are also widely used as aromatic agents in bread, cheese, and when preparing food due to their sweet scent [27,28,30].

The ND seeds contain essential oils (0.13–0.39%), alkaloids, sterols, phenolic compounds, saponins, fatty oils (36–50%), and proteins [7,21,22,29,30]. Several studies have been carried out on essential and vegetable oils of *Nigella* species constituents and their beneficial health properties [3,7,31,32,33,34,35]. However, the literature data on the nutritional composition of NS seeds are very limited and no information is available concerning the proximate composition and nutritional content of ND seeds. Furthermore, much less attention has been given so far to this species regarding its polar extracts, especially the phenolic fraction, as well as its biological properties. On the other hand, to the best of our knowledge, the studies investigating the comparison between NS and ND seed extracts regarding their phenolic profile and antioxidant activities are scarce in the literature and only Toma et al. [20] achieved work in this line. Furthermore, no study has examined the electrochemical measurements of antioxidant activity (voltametric method) for both species.

The present research aimed to explore and valorize the seeds of these species mainly in the pharmaceutical and food industries by assessing their principal biological effects in vitro and in vivo. It noteworthy that their chemical composition could also be investigated and compared. since it is mostly involved in these activities.

Therefore, in this current study, we attempt to compare the phenolic profiles of NS and ND seed extracts by the determination of total phenolic, total flavonoid, and tannin contents, as well by identifying the individual phenolics by high performance liquid chromatography with diode array detection (HPLC-DAD). Furthermore, the in vivo and in vitro antioxidant and antimicrobial properties of these extracts were assessed. Additionally, to overcome the lack of data about the proximate composition of the seeds of NS and ND, some nutritional constituents, including carbohydrates, proteins, fats, and mineral levels were also evaluated. Beforehand, to facilitate the botanical discrimination of the studied species, macroscopic and microscopic parameters were determined for the different species parts.

## 2. Results

### 2.1. Botanical Study

There is a great resemblance between the two investigated species of the genus *Nigella,* which can lead to confusion in their identification. To this end, a botanical study was carried out in this work to distinguish unambiguously between them. The macroscopical characters of NS and ND seeds are shown in Table 1.

**Figure 1:**
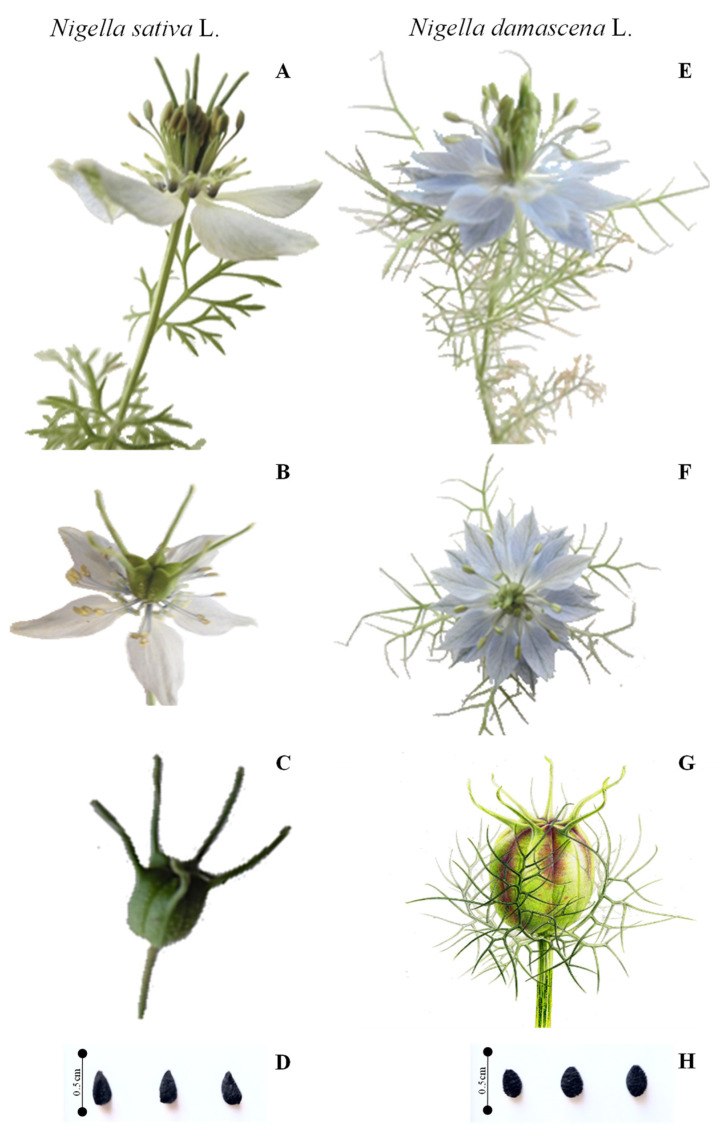
Photographs of aerial parts, flowers, fruits, and seeds of *Nigella sativa* L. (**A**–**D**) and *Nigella damascena* L. (**E**–**H**).

According to the main morphological parameters, NS can be recognized by the nonexistence of leaves united in involucre immediately around the flowers, which are light blue or white, sometimes slightly mixed with green. The leaves are divided into narrow and elongated lobes, often a little widened at the top. The fruit is a slightly rough capsule, and it is longer, green, and turns brownish when ripe. The seeds are small and black, with a hard tegument, and have a pleasant smell of camphor with a slightly bitter taste (Figure 1A–D).

The ND is easy to distinguish by its leaves, which form an involucre immediately around the flower or fruit, and by its pretty flowers of blue or white, each surrounded by the long and thin green strips of the involucres, which are longer than the sepals. The leaves are divided into sharp, narrow strips, similar to those of involucres. The fruit is a swollen and smooth capsule, which is oval-globular. The seeds are small and black, rounded and streaked (Figure 1E–H), their aroma slightly resembles the strawberry, and their taste is slightly bitter. Moreover, ND produced a larger plant and a higher number of capsules per plant compared to NS. These results were in agreement with those of Zaitoun et al. [36].The first detailed botanical study on 10 *Nigella* species was carried out by Kökdil et al. [37], who observed that the herbaceous stems are glabrescent or that glucose is found in most species with pinnatipartid or pinnatisect leaves, and that inflorescence has various characteristics.

Microscopical characteristics of seeds were also determined, and the results of SEM examination showed similarities and distinct variations between the seed shape, cells of reticulation, bounded area, and lumen floor, which is in accordance with the studies of some authors [38,39].

**Figure 2:**
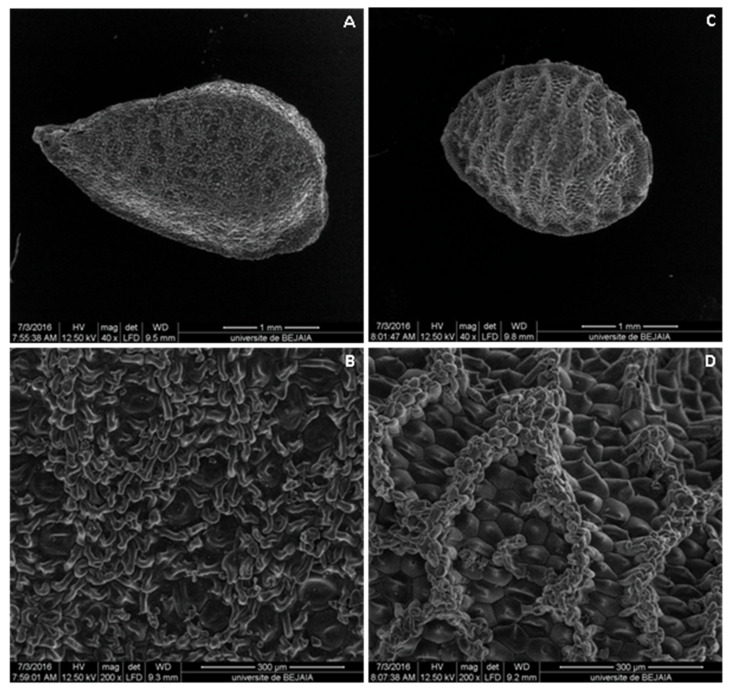
Scanning electron microscopy of seed surfaces. (**A**) General view of *Nigella sativa* L. seed; (**B**) closer view of *Nigella sativa* L. seed coat ornamentation, columellated and uncolumellated testa cells; (**C**) general view of *Nigella damascena* L. seed; (**D**) closer view of *Nigella damascena* L. seed coat ornamentation, cells of lumen and muri.

The NS seeds are columellated and have three high longitudinal ridges on each angle. The testa is composed of columellated and uncolumellated cells that are chattered on the seed as a single cell or a group of one to three among the columellated cells (Figure 2A,B). Otherwise, ND has a triquetrous to subpyramidal structure and reticulated seeds, as was already described by Dadandi et al. [39]. This reticulate structure is composed of three prominent lengthwise striations on each edge and cross continuous striations that are connected with them and each other. Striations are composed of rounded cells, which are sometimes slightly elongated, compressed from both sides to resemble erythrocytes. They are smaller than the lumen cells and are piled up irregularly. Lumens cells (hexagonal cells) are located inside of the reticulation and are characterized by the consistent presence of a tapering mucro at the center of cells (Figure 2C,D).

### 2.2. Chemical Composition

#### 2.2.1. Nutrient Contents

The proximate and mineral composition of NS and ND seeds are shown in Table 2. A significant difference (*p* ≤ 0.05) was found between the majority of nutrient contents of the two species. Total fats represent the key components in both species of seeds, followed by carbohydrates, crude proteins, and then reducing sugars, ashes, and moisture. The crude fats content was higher in NS seeds (42.30 ± 0.67%) than in ND ones (34.30 ± 0.35%), and the same was true for reducing sugars and moisture. Nevertheless, the opposing trend was noticed for crude proteins, total carbohydrates, ashes, and sodium quantities, where ND seeds hold the best quantities.

According to the literature, the nutritional value of NS is relatively good [26], but differences were observed in the measured contents. The estimated fats content was inferior to that presented by Kabir et al. [24], whereas carbohydrates were higher than the values presented by Kabir et al., and protein content was also relatively superior to that reported by Al-Jassir [12], Kabir et al. [24], and Barkah et al. [40]. It should be noted that no data are found about the proximal composition of ND seeds, and only a few existing studies have given crude fat and protein concentrations. Accordingly, Datta and Saha [38] recorded the best protein amount in ND seeds. These discrepancies in nutrient content may be due to variations in environmental factors, such as the geographic and climatic zones where the *Nigella* plants were grown [12,25].

#### 2.2.2. Phenolic Compound Contents

In addition to their richness in nutrients which represents the primary metabolites, the two investigated species are a noticeable in terms of their contents of secondary metabolites, including phenolic compounds. The ND seeds contained significantly (*p* ≤ 0.05) higher total extractable compounds and phenolic contents (TPC, TFC, and tannins) than NS seeds (Table 3). These results are in agreement with those reported in the literature [20,41].

Lower amounts of total phenolics in NS seeds adopting various extraction techniques and solvents were reported in several studies. Thippeswamy and Naidu [42] recorded content of 410 mg GAE/100 g DW (methanol 80%, Soxhlet extraction), and Khattak and Simpson [43] found 410 mg GAE/100 g DW in the non-irradiated methanolic extract by using Soxhlet extraction either combined or not combined with gamma-irradiation. Nevertheless, the highest contents of TPC were evidenced by Al-Bishri and Danial [44] which were 1580 mg GAE/100 g DW (ethanol 95%, maceration).

Regarding total flavonoid contents, Javorková et al. [41] recorded higher values with 500 and 2100 mg/100 g DW in NS and ND extracts, respectively.

However, data of the two *Nigella* species are difficult to compare with previous works due to different expressions regarding the referring standard and used material basis. Indeed, caffeic acid (CAE) and rutin equivalents (RE) for NS (412 mg CAE/100 g dry extract, and 200 mg RE/100 g dry extract, respectively) and for ND (2383 mg CAE/100 g and 1453 mg RE/100 g, respectively) were used by Toma et al. [20] for expressing the results of TPC and flavonoids, respectively. In the same line, when Dalli et al. [45] expressed their results referring to extract basis, they obtained 3100 mg GAE/100 g and 1840 mg QE/100 g for phenolic and flavonoid contents, respectively.

Several factors can cause dissimilarities in phenolic contents, such as genetic factors [46,47], ripening region [48], and parameters related to the extraction method (temperature, time contact, solvent-to-solid ratio, extraction method, solvent used, etc.) [43,49,50]. The biotic (pathogens and herbivores) and abiotic factors (climatic conditions, soil composition, altitude, drought stress, salinity) [48,51,52] could also be implicated. Indeed, Navarro et al. [48] and Shahbazi et al. [7] reported that water stress enhances the production of oxygen species and free radicals in plants, which increased biosynthesis and the accumulation of phenolic compounds as a plant protection mechanism. Thus, Shahbazi et al. [7] noted very important amounts of TPC (6000 mg GAE/100 g DW in NS and 6300 mg GAE/100 g DW in ND), and TFC (2400 mg QE/100 g DW for both species) under drought stress during two growing seasons.

#### 2.2.3. Identification and Quantification of Phenolic Compounds Using HPLC-DAD

**Figure 3:**
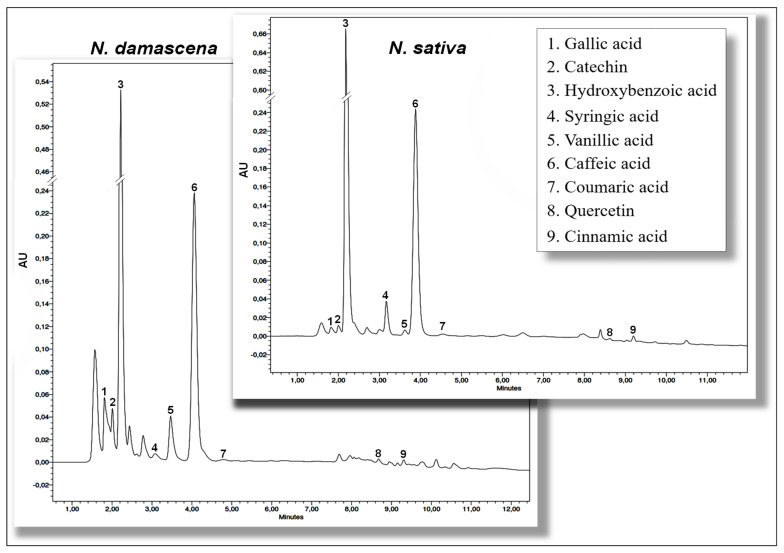
HPLC/DAD chromatographic profiles of *Nigella damascena* L. and *Nigella sativa* L. extracts monitored at 280 nm.

Qualitative and quantitative analyses of polyphenols in NS and ND methanolic extracts were performed by HPLC-DAD. The chromatograms resulting from both species have almost the same profile (Figure 3), and similar phenolic derivatives were detected with a difference in the amounts of constituents. According to the results (Table 4), hydroxybenzoic and caffeic acids were the major phenolic compounds of both species. They were followed by vanillic, syringic, gallic, and *p*-coumaric acids, and catechin. Nevertheless, low amounts of cinnamic acid and quercetin were noticed. Still, ND seed extract contains higher amounts of individual phenolic compounds than NS extract.

Data concerning the HPLC-DAD phenolic profiles of the studied species are rather scarce in the literature, mainly for ND extract, and revealed a discrepancy in the results of their qualitative and quantitative phenolic composition, especially the major compounds. Indeed, in the study of Toma et al. [20], the same phenolic compounds were identified in the hydrolyzed extracts of NS and ND, and the most dominant compounds were kaempferol and ferulic acid, but glycosylated flavonols (quercitrin and hyperoside) were also determined before hydrolysis. Topcagic et al. [5] identified sinapinic acid and kaempferol as the main phenolic compounds in NS seed extracts and revealed that the majority of phenolic acids were bound to cell walls in ester or glycosylated forms. Moreover, Toma et al. [20] suggested the presence of conjugated glycoside compounds in non-hydrolyzed extracts of NS and ND seeds. This could explain the presence of significant amounts of phenolic acids in the extracts of the present study, which could be obtained after hydrolysis of more complex esterified or glycosylated compounds, such as gallotannins and flavonoid glycosides. Indeed, Merfort et al. [53] isolated three flavonol triglycosides of quercetin and kaempferol from NS seeds, and Fico et al. [29] isolated phenolic ester (1-O-2,4-dihydroxy phenylacetyl glycerol) from ND seeds. In NS seedcake extracts, p-coumaric acid was the dominant constituent followed by hydroxybenzoic acid [54], while in another study gallic acid and rutin were the major compounds [55]. More recently, researchers have confirmed the presence of gallic acid, hydroquinone, rutin, quercetin, and kaempferol in NS extracts [56], while high amounts of dihydroxybenzoic and ferulic acids were found by Ansary et al. [57].

The differences observed in the phenolic characterization of the two species and those of previous research could be attributed to numerous factors. In fact, the metabolism of phenolic compounds in plants is very complex, and the phenolic profiles varies considerably between different organisms depending on various conditions, such as the extraction solvents used, extracts’ nature (hydrolyzed or not) [20], stage of plant development, genetic factors, agronomic practices, and climatic and stress conditions [5,13,57,58,59].

### 2.3. Biological Activities

#### 2.3.1. In Vitro Antioxidant Activities

The antioxidant properties of plants are mainly due to their redox potential, including hydrogen donors, reducing agents, singlet oxygen quenchers, and metal chelators [55,60,61]. In this study, numerous in vitro antioxidant tests were carried out to assess the antioxidant activities of NS and ND seed extracts, namely the reducing power, free radical scavenging tests (DPPH^•^ and ABTS^•+^ radicals), and an electrochemical technique (based on the reactivity of electrogenerated superoxide). This developed method [62,63] was investigated in *Nigella* extracts for the first time, compared with conventional spectrophotometric antioxidant tests.

In iron reducing power, NS and ND extracts demonstrated the ability to reduce ferric (Fe^3+^) to ferrous (Fe^2+^) ions. Thus, results showed that ND seeds extract exhibited a higher reducing effect than NS. Whatever gallic acid and quercetin were used as references, antioxidants presented the most pronounced capacity (Table 3). Conversely, Toma et al. [20] indicated that NS seeds exhibited better iron reduction capacity than ND seeds, although ND contained higher amounts of TPC and TFC. Conversely, a lower reducing capacity for NS was indicated by Dalli et al. [45], even though, as mentioned earlier, the amounts of total phenolics and flavonoids found in their study were much higher. This can be attributed to the nature of the extracted compounds. Thus, they demonstrated that the various types of extracts do not contain the same type of molecules and, therefore, cannot have a comparable iron-reducing effect [45].

With regard to scavenging activity against the DPPH^•^ radical, the two species were able to trap this radical, but ND displayed a more elevated impact than NS; the concentrations of both extracts necessary to scavenge 50% of the radical (IC_50_) were 318.84 ± 0.02 mg/L and 617.52 ± 0.01 mg/L, respectively. This activity remains less than that of the reference antioxidants used (Table 3). Higher anti-DPPH^•^ activity was obtained by Toma et al. [20] for ND seeds, with an IC_50_ of 178 mg/L, but a lower capacity was found for NS with an IC_50_ of 625 mg/L. Recently, a very potent anti-DPPH^•^ effect was reported for NS (IC_50_ = 2.43 mg/L) and ND (IC_50_ = 2.51 mg/L) from Iran cultivated under drought stress during two growing seasons [7].

The results of scavenging activity towards ABTS^•+^ were in accordance with those of DPPH^•^ (Table 3), revealing that ND seeds exhibited a significantly (*p* ≤ 0.05) higher scavenger capacity with lower concentration of the extract required to scavenge 50% (IC_50_) or 30% (IC_30_) of the radical (IC_50_ = 44.15 ± 0.02 mg/mL and IC_30_ = 0.016 ± 0.001 mg/mL). However, this scavenging effect was lower than that of Trolox^®^, quercetin, and gallic acid employed as reference molecules. There is little data comparing the ABTS^•+^ scavenging activity of NS and ND extracts; only Toma et al. [20] showed that ND had a higher ABTS^•+^ scavenging capacity than NS, and this scavenging effect was higher than that recorded in this current study.

**Figure 4:**
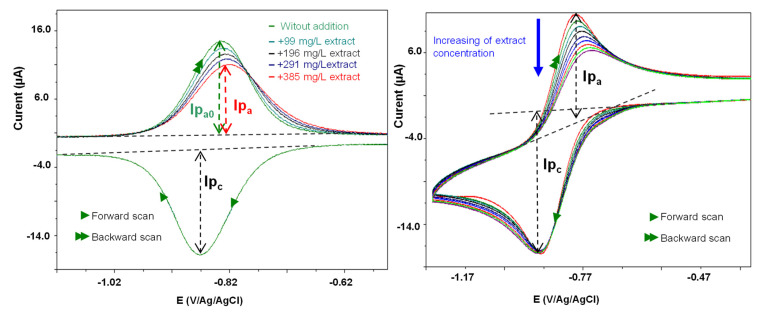
Cyclic voltammograms of O_2_^•−^ in the absence and the presence of increasing concentrations of *Nigella damascena* L. extract at a steady glassy carbon disk electrode in DMF/0.1 M Bu_4_NPF_6_; scan rate 0.1 V ^s−1^. (**A**) Time semi-derivative convoluted curves; (**B**) CV curves.

The scavenging activity towards electrochemically generated O_2_^•−^ determines the decline of the oxidation peak current value of the O_2_^•−^ generated in cyclic voltammetry, electrochemically, from the dissolved oxygen reduction. This technique has been extended successfully to seaweeds [62,64], *Mentha* [65], and *Erica* [66] plant extracts. However, the manner to detect the peak currents was different from the method previously reported. Thus, for the measurements of these peaks, the cyclic voltammogram (CV) exploitation was carried out using the convolution time semi-derivative transformation [67]. Figure 4 shows the cyclic voltammograms of O_2_^• −^in the absence and presence of increasing concentrations of ND. The O_2_^•−^ scavenging power was higher for ND extract (IC_50_ = 854.3 mg/L) than for NS (IC_50_ = 994.73 mg/L). These antioxidant index values (O_2_^•−^ quenching) correlate with IC_50_measured in the DPPH^•^ and ABTS^•+^ tests and without interference in turbidity or colored extract solutions in the electrochemical technique (Table 3).

It should be noticed that some powerful antioxidants and suppressors of oxidative stress were identified in both extracts, such as hydroxybenzoic, gallic, caffeic, syringic, coumaric acids, and catechin [68,69]. Thus, a correlation between the phenolic contents (TPC, TFC, and tannins) and the antioxidant properties of both species could be evident, indicating that ND seeds possess a significantly higher antioxidant capacity. Shahbazi et al. [7] stated a correlation between TPC and TFC contents with the scavenging capacity of DPPH^•^. Toma et al. [20] reported the same correlation except for the ferric reducing power. This implies that the in vitro antioxidant effect (radical scavenging activity and ferric reducing ability) of NS and ND seed extracts depends on the quality and quantity of their phenolics.

#### 2.3.2. In Vivo Antioxidant Activity

The in vitro antioxidant activity estimated by several chemical and biochemical assays cannot, reasonably, be used to suggest that the electron-donating phenolic compounds can stimulate the antioxidant defense of the organism [68]. Nonetheless, owing to their abundance in plant food, the corresponding phenolic products could be important micronutrients for fighting oxidative stress in the stomach [70]. Thus, the assessment of in vivo antioxidant capacity for NS and ND extracts is of critical relevance. It was achieved by the feeding mice technique, where different analyses were exploited, namely blood collection, plasma antioxidant capacity, and serum biochemistry.

**Figure 5:**
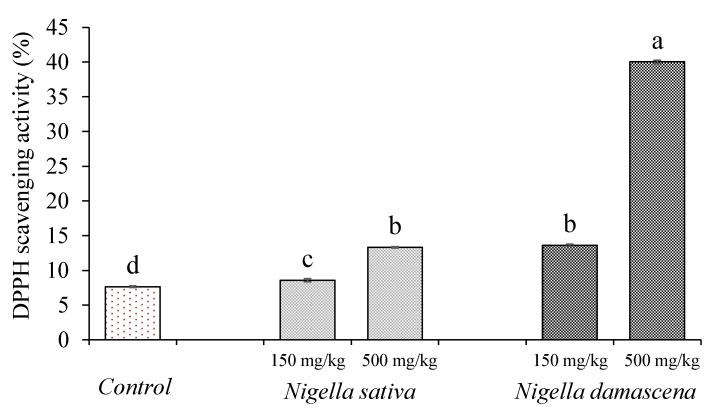
Plasma antioxidant capacity toward DPPH radical for different mice groups. Values are the means ± SEM. Results with different letters are statistically different (Test-LSD, *p* < 0.05, a > b > c > d). Control, group of mice treated with the 0.9% saline solution; *Nigella sativa*, two groups of mice treated with the extract of *Nigella sativa* L. (150 mg/kg body weight for NS1 and 500 mg/kg body weight for NS2); *Nigella damascena*, two groups of mice treated with the *Nigella damascena* L. extract (150 mg/kg of body weight for ND1 group and 500 mg/kg of body weight for ND2 group).

For the assessment of the efficiency of antioxidant treatment or dietary supplementation, plasma antioxidant capacity (PAC) is one of the most widely employed biomarkers. In this study, NS and ND extracts were administered orally on Swiss albino mice for 3 weeks, and their effect on oxidant/antioxidant balance and biochemical parameters were investigated. The administration of ND extract (500 mg/kg) for the group ND2, leads to a significantly (*p* ≤ 0.05) higher increase in PAC (40.03 ± 0.95%) versus the NS extract (13.62 ± 0.15%) and control group (7.65 ± 0.13%). Although, a non-significant (*p* ≤ 0.05) increase in the PAC in the control group was observed during the administration of a lower dose (150 mg/kg) for the group NS1 (8.57 ± 0.24%), this antioxidant capacity was still significantly (*p* ≤ 0.05) inferior, compared with the ND1 group (13.31 ± 0.15%) (Figure 5).

Similar results regarding NS extract were reported by Meziti et al. [71], using methanolic extract and testing 500 mg/kg dose (49.62 ± 2.26% versus 26.34 ± 6.14% for the control group). The elevated plasma antioxidant capacity might be ascribed to the increased level of exogenous antioxidants acquired after treatment with methanolic seed extracts of ND and NS. Indeed, the plasma contains different endogenous antioxidants (uric acid, albumin bilirubin, reduced glutathione) and dietary exogenous antioxidants [68], that may act in a synergistic manner to improve protection against reactive oxygen species (ROS).

The biochemical parameters of the orally administered *Nigella* extracts decreased significantly (*p* ≤ 0.05) compared to control mice (Table 5), while the best results were obtained at 500 mg/kg body weight. Thus, ND extract resulted in a significant reduction (*p* ≤ 0.05) in protein and albumin levels, which are related to liver functionality, blood glucose, urea, and plasma lipid profile (cholesterol and triglycerides), in comparison with the control and NS1 groups of the animals. It is noteworthy that all groups present a high level of blood glucose, but it was lower in animals treated with *Nigella* methanolic extracts, namely ND2, which significantly (*p* ≤ 0.05) brought down glycemia (1.58 ± 0.02 g/L versus 2.50 ± 0.35 g/L for the control group). However, a slight decrease in blood glucose was observed when NS1 extract was administered (2.15 ± 0.63 g/L), but this level was significantly (*p* ≤ 0.05) lower than in the control mice.

The current investigation confirms previous studies which showed that NS seed extract affects biochemical and blood parameters [72,73]. Kaleem et al. [73] reported that oral administration of ethanolic extract of NS seeds (300 mg/kg) to diabetic rats for 4 weeks significantly (*p* ≤ 0.05) decreased their high levels of blood glucose and plasma lipids. Treatment of diabetic rats with methanol extract of NS for 30 days also showed a significant (*p* ≤ 0.05) reduction in glucose rate [72].

In addition, the powder of NS seeds at 5% enhanced the lipid profile in hypercholesterolemic rabbits, with a significant (*p* ≤ 0.05) reduction in total cholesterol and low-density lipoprotein (LDL) [74]. Similarly, a study carried out on mice fed with a high fat diet and hydroalcoholic NS extract (200 mg/kg), found that the total cholesterol level was significantly (*p* ≤ 0.05) decreased [75]. Another study also suggested that the treatment of diabetic rats with an ethanolic extract from NS (100–400 mg/kg) for 40 days decreased plasma glucose and lipids [76].

Previous research demonstrated the involvement of several pathways in the effect of the NS extract on biochemical parameters. According to Kanter et al. [77], NS exhibits a protective effect in the diabetic rat by preserving insulin-producing pancreatic cells and inhibiting the oxidative stress in the cells. The ethanolic extract of NS seeds has revealed in vitro hypoglycemic activity in cells using adipocytes [78], by activation of the AMP-activated protein kinase (AMPK) pathway and the insulin signaling pathway, in addition to its agonistic role in the peroxisome proliferator-activated receptor (PPARγ), which has a function in metabolic regulation [79]. Moreover, the antidiabetic properties of aqueous crude extract of NS directly inhibit the intestinal absorption and enhancement of carbohydrate tolerance [80].

Furthermore, NS could play a role in the regulation of serum lipid profile and, therefore, offer cardioprotective properties. An inhibitory effect on the heart rate and contractility was found after regular intake of NS aqueous extract. This effect was attributed to the opening of potassium channels [81]. Furthermore, in vivo oral intake of ethanolic extract of NS seed (400 mg/kg) lowered the lipid peroxidation in Wistar rats. Another mechanism was described where a lowered serum cardiac troponin-Y, high sensitivity C-reactive protein (hsCRP), and lysosomal thiobarbituric acid reactive substances (TBARS) were reported [82].

Assessment of the serum total protein and albumin provides information on the liver functionality, and their reduction indicates decreased synthetic function, evident in liver damage or diseases, whereas a rise of these parameters is mostly observed in cancerous illnesses or through a high protein diet [83,84].

The measurement of the urea plasma level can give an insight into the function of the kidneys. An elevated blood rate in urea depicts kidney dysfunction, or that the animal is dehydrated; however, reduced urea levels are associated with overhydration or acute liver failure [84,85]. In the present work, mice treated with the two species of *Nigella* seed extracts revealed enhancement in some parameters (serum total protein, albumin, and urea), indicating possible prevention of liver and kidney complications.

To the author’s knowledge, there are no data available in the literature on the biochemical effects of exploring the in vivo antioxidant activity of ND crude extract. An investigation was reported on the acute and sub-chronic toxicity of this species which described no significant (*p* ≤ 0.05) difference concerning clinical blood chemistry examination (glucose, cholesterol, urea, total protein, and albumin) after oral gavages of mice by seed methanolic extract (100–400 mg/kg), and it exhibited no symptoms of toxicity [86].

#### 2.3.3. Antimicrobial Activity

According to the results of the antimicrobial activity of NS and ND extracts (Table 6), an elevated concentration of 195 mg/mL was necessary to exhibit an activity towards the two tested Gram-positive strains. A better activity was reported for ND extract towards methicillin-sensitive *Staphylococcus aureus* (MSSA) with an inhibition diameter of 22.0 ± 0.5 mm. However, NS extract showed only a diameter of 14.0 ± 0.5 mm. Interestingly, both extracts demonstrated similar activity against methicillin-resistant *Staphylococcus aureus* (MRSA) with a diameter of 15.0 ± 0.3 mm. Otherwise, NS extract has no activity against the Gram-negative bacteria, for both concentrations used, and ND extract has only an appreciable activity against *Pseudomonas aeroginosa* (18.0 ± 0.5 mm).

The found results can be explained by the fact that Gram-positive isolated bacteria were usually affected more than Gram-negative ones [87,88,89]. This result could be linked to the lack of an outer membrane of lipopolysaccharides and lipoproteins in Gram-positive bacteria, which acts as a barrier to bioactive molecules contained in the extracts [88,89].

Regarding antifungal activity, NS extract had an effect against *A. flavus* (16.0 ± 0.4 mm), *M. rammanianus* (17.0 ± 0.4 mm), *A. parasiticus* (17.0 ± 0.3 mm), and *C. albicans* (13.0 ± 0.5 mm), but no activity against *A. ochraceus* and *A. niger.* The ND extract demonstrated an effect against the four studied strains, with better activity against *A. parasiticus* and *A. flavus,* with inhibition diameters of 22.0 ± 0.5 and 20.0 ± 0.5 mm, respectively. However, this extract had no activity towards *A. niger* and *C. albicans*. It is quite clear that it was the extract of ND, which was more active than that of NS except against *C. albicans*. The seed extracts of NS [17,87,90,91] and ND [27,91] have previously shown important antimicrobial activity. However, it is substantial to highlight that the number of studies related to ND was limited.

Both plants are generally considered as the most pharmacologically active species of the genus *Nigella* [91,92] but the results obtained by Kokoska [2] showed that extracts from *N. arvensis* and *N. orientalis* seeds were more effective. The study of Hanafy and Hatem [87] showed that discs impregnated with the diethyl ether extract of NS seeds (25–400 µg extract/disc) caused inhibition of *S. haureus*, *E. coli*, *P. aeruginosa*, and *C. albicans*, but not *S. typhimurium*. The diameter of the zone of inhibition was proportional to the logarithm of disc drug content, as the studies of Hanafy and Hatem [87], Fico et al. [27], and Hasan et al. [93] showed that there is a significant (*p* ≤ 0.05) correlation between inhibition zones and extract concentrations.

According to Bakathir and Abbas [94], the inhibition of the growth of *S. aureus* was observed at a concentration of 300 mg/mL using water crude extract of NS seeds. No inhibition was found in the growth of *E. coli* and *Enterobacter*. Hasan et al.’s [93] analysis showed that methanolic extract of NS exhibited significantly higher antibacterial activity at the concentration of 50 mg/mL (*p* ≤ 0.01) against *S. pyogenes*, with a greater inhibition zone of 19 mm, while an inhibition zone of 15 mm was observed against *P. aeruginosa*, *K. pneumonia*, and *P. vulgaris*. These data corroborate generally to our results, but the absence of activity towards Gram-negative bacteria by testing NS may be essentially due to differences in the extraction parameters, the solvents, and the extract concentrations used. Some authors revealed comparable results to those of this current study regarding the antibacterial activity of ND seed extracts. Fico et al. [27] showed that the BuOH extract of ND inhibited the growth of *P. aeruginosa* (MIC = 2.25 mg/mL) and *S. aureus* (MIC = 1.125 mg/mL).

Among the four *Nigella* species including NS and ND explored by Tanis et al. [91], acetonic extract of NS seeds was the most effective on both Gram-positive and Gram-negative bacteria but least effective against *K. pneumonia,* which was not in line with our results. This can be explained by the fact that the extracted product varied in terms of quality, quantity, and composition according to the extraction parameters, the solvent used, plant organ, age, climate, soil composition, etc. [91,95].

There is little data on the effect of NS and ND extracts on fungi strains. The studies of Mahmoudvand et al. [96] and Sheik Noor et al. [97] demonstrated that NS extracts and their essential oil possess remarkable antifungal and antidermatophytic activities. The results of Ariamanesh et al. [98] study confirmed the effectiveness of NS alcoholic extract on the inhibition of *C. albicans* colonization and formation of plaque on the acrylic denture, and this effect was dose-dependent. The anti-yeast activity of NS seeds extracts has been also studied by Nadaf et al. [90], and it was observed that this extract showed an effect against *C. albicans, S. cerevisiae*, and *C. utilis*. This outcome was detected by scanning electron microscopy, which revealed that the treated yeast cells showed abnormal morphologies, such as injuries on the cell surface and denatured cells. The extract’s phytochemicals may interfere with cell wall synthesis of the budding yeast and engender some cytotoxic activities by cell membrane disruption followed by leakage of cytoplasmic constituents, as well as inhibition of intercellular and extracellular enzymes synthesis [90,99].

Among these phytochemicals are found phenolic compounds (flavonoids, isoflavonoids, and tannins) alkaloids, glycosides, and terpenes [100]. Hence, the studies of Hanafy and Hatem [87], Halawani [101], and Bakathir and Abbas [94] demonstrated that NS antimicrobial activity may be attributed to the mixture of its phenolic compounds and important active ingredients including thymoquinone, thymohydroquinone, and melanin. Additionally, phenolic compounds, such as kaempferol, caffeic, and coumaric acids could be responsible for the antibacterial activity of ND seed extracts; a synergy between these phenolics and other compounds present in this extract could also have an impact [27]. Consequently, in this paper, a correlation between the phenolic components and the antimicrobial activity of NS and ND could be evident.

## 3. Materials and Methods

### 3.1. Plants Material

The two plants investigated in the present study *Nigella sativa* L. and *Nigella damascena* L. were procured from the Seddouk locality (Bejaia Department, Algeria) where they were sown at about 400 m of altitude. Their identification was initially carried out at the botanical laboratory of the Laboratory of Plant Biology, University of Bejaia, Algeria, and was then confirmed by Dr. Saladin (a lecturer in plant biology and plant physiology at Limoges University, France), before voucher specimens were deposited in the Faculty of Science and Technology of the Limoges University under the following references: *Nigella sativa* (Ns1.Ran.2022) and *Nigella damascena* (Nd3.Ran.2022). The differences in botanical features between the two species were summarized in Table 1 and the photographs of plants, flowers, and seeds of both plants were illustrated in Figure 1. The seeds were also observed by scanning electron microscopy (SEM) (Philips XL-30 FEG-SEM, Oslo, Finland), and their images were recorded at different magnifications (Figure 2).

The seeds were discarded and dried in an oven at 40 °C until mass stabilization, then ground (IKA A11 Basic, Berlin, Germany) and sieved (sieveshakerAS200basic, Retsch GmbH, Haan, Berlin, Germany) to a granulometry ≤ 500 µm. The obtained powders were adequately stored.

### 3.2. Proximal Analysis

The proximal analysis was conducted according to the Association of Official Analytical Chemists’ methods [102], as it has already reported on the determination of the nutritional composition of *Nigella sativa* L. seeds [12,24,25]. The determination of moisture of the seeds was measured by a thermogravimetric procedure at 105 °C. Total nitrogen was determined using the Kjeldahl method, and the estimation of crude protein concentration was expressed as 6.25 × N. Ash content was obtained after incineration of samples at 600 °C for 6 h. Minerals (sodium and magnesium) were determined using an atomic absorption spectrophotometer (Perkin-Elmer model 5000, Whaltam, MA, USA), and total fat content was obtained by the Soxhlet method using n-hexane as a solvent. Total carbohydrate content was deduced by the subtraction of all other compounds (crude proteins, ashes, crude fats, and moisture) from 100, and reducing sugars (oses and osides) were determined following the Bertrand method.

### 3.3. Phenolic Compounds Analysis

For the extraction of phenolic compounds, 100 g of seed powders were macerated using 1000 mL methanol–water (80/20, *v/v*) for 24 h at room temperature in the dark under magnetic stirring (Trade Raypa, AG-5, Analitica De Mori, Italia). The extract was filtered and concentrated in a ventilated oven at 40 °C (Memmert, Berlin, Germany). The remaining phase was treated with petroleum ether to remove lipids, and then lyophilized (Christ, Alpha 1-4 LD Plus, Osterode, Germany). The freeze-dried sample was reconstituted in methanol.

Total phenolic content (TPC) was determined using Folin–Ciocalteu reagent according to Swain and Hillis [103]. Total flavonoid content (TFC) was estimated by Bahorun et al.’s [104] method using aluminum chloride (AlCl_3_) as a reagent, and tannin amount was determined by protein-precipitation assay according to the method of Hagerman and Butler [105]. The TPC, TFC, and tannin contents were expressed as mg of gallic acid (GA), quercetin (Q), and tannic acid (TA) equivalents per 100 g of dry weight (DW) of plant, respectively.

The identification and quantification of the individual phenolic compounds of NS and ND were performed according to Bourgou et al. [106] with some modifications, using high-performance liquid chromatography (Waters 2690, Milford, CT, USA), with a devidce equipped with a Waters 996 Diode Array Detector (Waters 996, Milford, USA) (HPLC-DAD). Before analysis, the extracts were hydrolyzed under 6M HCl at 90 °C; the obtained mixture was filtered through a 0.22 μm Millipore membrane. Chromatographic separations were performed on a Kinetex Column (RP C18, 2.6 µm, 50 × 4.6 mm); the mobile phase consisted of acetonitrile (A) and water–acetic acid (94/6, *v*/*v*) (B). The elution gradient was as follows: 15–85% B in 7 min, 40–60 B in 3 min, 60–40% B in 2 min, 80–20% B in 2.5 min, and 100–0% B in 7.5 min. A flow of 0.3 mL/min was used, the injection volume was 10 μL, and the detection was carried out at 280 nm. Phenolic compounds were identified and quantified by comparing the retention times and peak areas with those of authentic standards, and all compounds were confirmed using internal standards.

### 3.4. Bioactive Properties

#### 3.4.1. In Vitro Antioxidant Activities

The reducing power of seed extracts was determined according to the method of Oyaizu [107], and results were expressed as A_0.5_ at 700 nm; gallic acid and quercetin were used as positive controls. Free radical scavenging activity was evaluated by DPPH^•^ (1,1-diphenyl 1-2-picrylhydrazyl) and ABTS^•+^ (2,2′-azino-bis(3-ethylbenzothiazoline-6-sulfonic acid)) [108] assays, and results were expressed as IC_50_ and IC_30_ (mg/L). Here, BHA, ascorbic acid, and quercetin were used as control standards for the DPPH assay, and Trolox^®^, quercetin, and gallic acid were used as references for ABTS one.

Antioxidant capacity was also determined by an alternative electrochemical method which was first developed by Le Bourvellec et al. [63] and applied for *Erica* [66] and *Mentha* [65] crude extracts. This method using the cyclic voltammetric technique, measures the reactivity of antioxidant compounds with electrogenerated superoxide radical (O_2_^•−^). The electrochemical measurements were carried out using a dual potentio/galvanostat PGSTAT 30 (Autolab Instruments, Eco Chemie B.V., Utrecht, the Netherlands), and the voltammograms were recorded via the GPES software (General Purpose Electrochemical System version 4.9, Eco Chemie B.V.). The results were estimated as the antioxidant index value recorded as IC_50_, and Trolox^®^, quercetin, and gallic acid were used as reference standards.

The cell consists of three electrodes, namely a glassy carbon disk working electrode (diameter 2 mm), with a platinum wire counter electrode and a reference electrode Ag/AgCl in EtOH. The reference electrode was separated from the solution by a salt bridge containing 0.5 M of Bu_4_NPF_6_. All the potentials are given relative to this electrode. The cyclic voltammogram of the oxygen reduction was then recorded at a scan rate of 0.1 Vs^−1^, via a two-way scan between 0 and −1.5 V vs. Ag/AgCl reference electrode. Aliquots of crude extracts were successively added to the 10 mL oxygen solution. After each aliquot addition, the CV of the oxygen solution was recorded at a scan rate of 0.1 Vs^−1^ (see registered voltammograms in Figure 4). The measurement of the antioxidant activity is estimated by the antioxidant index value. Here, IC_30_ or IC_50_, defined as the phenolic compound or extract concentration needed to consume, respectively, show 30% or 50% of the electro-generated radicals [corresponding to (Ipa0 − IpaS)/Ipa0 = 0.3 or 0.5 where Ipa0 is the intensity of the anodic current peak of O_2_^•−^ and IpaS the intensity of the anodic current peak of O_2_^•−^ for the concentration S of the sample]. With this characterization, the lower the IC_30_ or IC_50_ value, the more the substrate has a strong reactivity toward the superoxide.

#### 3.4.2. In Vivo Antioxidant Properties

The assessment of in vivo antioxidant capacity was determined using the method proposed by Hasani et al. [109] and applied for NS extract by Meziti et al. [71]. Swiss albino male mice, weighing from 25 to 30 g, were obtained from the Pasteur Institute of Algiers (Algeria). There were kept under standardized conditions (temperature of 25–30 °C and a light/dark cycle of 12/12 h). The animals were fed with a standard diet (National Office of Cattle Feed of Bejaia, Algeria) and water ad libitum. The in vivo experiments were carried out after being approved by the Ethics and Deontology Committee of the University of Bejaia, Algeria, according to directive N° 2010/63/EU (22/09/2010) of the European Parliament and the Council of the European. Union.

After one week of acclimatization, mice were divided into five groups, each including five animals. Lyophilized extracts of NS and ND were dissolved in saline solution (NaCl 0.9%). Different obtained solutions (250 μL) were administered by gavages for *each* mouse daily for three weeks as follows:

(1) was the control group of mice treated with the saline solution (CTL); (2) was the group of mice treated with NS extract at 150 mg/kg body weight (NS1), (3) was the group of mice treated with NS extract at 500 mg/kg body weight (NS2), (4) was the group of mice treated with ND extract at 150 mg/kg body weight (ND1), and (5) was the group of mice treated with ND extract at 500 mg/kg body weight) (ND2).

The plasma antioxidant capacity (PAC) was evaluated at the end of the experimental period; blood samples were collected through direct heart puncture from anesthetized mice. Blood was recovered in heparinized tubes and subsequently centrifuged at 2000× *g* for 10 min to separate serum, which was collected in Eppendorf tubes for the determination of antioxidant status. The PAC was estimated by the DPPH^•^ radical scavenging assay according to Mansouri et al. [110], and results were expressed as percentages.

To determine the effects of *Nigella* extracts on the blood of mice, biochemical compositions were carried out on serums. Therefore, the medical laboratory’s internal protocols were used to determine total protein, albumin, glycemia, triglycerides, urea, and cholesterol concentrations.

#### 3.4.3. Antimicrobial Activity

For the determination of the antimicrobial activity of NS and ND extracts, the following 10 microbial strains were used: *Escherichia coli* (ATCC 25922) and *Pseudomonas aeruginosa* (ATCC 27853) were used as Gram-negative strains; methicillin-resistant *Staphylococcus aureus* (MRSA) (ATCC 43300) and methicillin-sensitive *Staphylococcus aureus* (MSSA) (ATCC 29213) were tested as Gram-positive strains. For the determination of antifungal activity, *Aspergillus niger* (939N), *Aspergillus flavus* (NRRL 3251), *Aspergillus parasiticus* (CB5), *Aspergillus ochraceus* (NRRL 3174), *Mucor rammanianus* (NRRL 1829), and *Candida albicans* (ATCC 10231) were used.

The antimicrobial action was carried out using the agar well diffusion method of Voidarou et al. [111]. The NS and ND dry extracts were prepared in DMSO (dimethyl sulfoxide) at two concentrations (C_1_: 5 mg/mL) and (C_2_: 195 mg/mL). For each Petri dish, wells of 9 mm in diameter were made and filled with 100 µL of each sample concentration, then incubated at 37 °C for 24 h for bacteria and 48 h at 30 °C for fungi and yeast strains. The antimicrobial activity was expressed as the diameter of the inhibition zones (mm), produced around the wells.

### 3.5. Statistical Analysis

All experiments were conducted in triplicate and results were expressed as mean ± standard deviation. The multiple means comparison was performed by analysis of variance (ANOVA), and the comparison of two means was conducted through a Student’s *t*-test. All statistical analyses were carried out with Statistica 5.5 software, and significance was taken at a level of *p* ≤ 0.05.

## 4. Conclusions

In this study, a botanical distinction by the determination of macro-morphological characteristics was evidenced; ND was characterized by involucrate blue flowers with 5–25 sepals. A difference was also noticeable in the seeds of the two species; ND seeds are smaller, more ridged, more rounded, and have a sweet scent, slightly resembling those of strawberry, but NS seeds have a pleasant smell of camphor with a slightly bitter taste. Regarding the microscopic evaluation, NS seeds have a testa, which is composed of columellated and uncolumellated cells, while ND seeds have lumens cells (hexagonal cells), which are located inside of that reticulation. Furthermore, it can be concluded that NS and ND seeds are a good source of fats, proteins, and carbohydrates, which can be used as an energetic food or as food supplements. The studied seeds are also an important source of phenolic compounds, namely hydroxybenzoic, caffeic, vanillic, and coumaric acids and it is noted that the seeds of *Nigella damascena* L. were richer in terms of phenolic composition than *Nigella sativa* L. seeds. However, this difference was more quantitative than qualitative. In addition, it would be interesting to carry out a more detailed study to compare the phenolic contents of the two *Nigella* species and analyzing the extracts using advanced techniques, such as ultra high-performance liquid chromatography and mass spectrometry (UHPLC-MS/MS). Interestingly, the evaluated plants demonstrated various antioxidant effects with a noticeable activity assigned to ND in the different assays. It is also worth mentioning that this species has revealed an interesting effect in the electrochemical method that was carried out for the first time on the *Nigella* genus. During in vivo assay, the same species revealed enhancement in some parameters, namely serum total protein, albumin, and urea, indicating a possible prevention of liver and kidney complications. On the other hand, it is noteworthy that the investigated extracts have an interesting hypoglycemic and hepatoprotective effects that could be explored further. Both species also demonstrate substantial vegetable oil contents, which might be implicated in the explored activities, and their comparison with the extract will be advantageous. Furthermore, the majority of the microbial strains including, *Pseudomonas aeruginosa*, *Staphylococcus aureus* Methicillin sensitive, *Aspergillus flavus,* and *Aspergillus parasiticus*, were sensitive to the ND extract. Overall, this study justified the large use of the *Nigella* species in traditional medicine. As such, both the studied plants, especially ND, could be considered as potent pharmacologically active species that promote their applications in different fields, including the pharmaceutical, cosmetics, and food industries.

## Figures and Tables

**Table 1:** Morphological characters of *Nigella sativa* L. and *Nigella damascena* L. seeds.

Parts	*Nigella sativa* L.	*Nigella damascena* L.
Stem	Erect, ribbed, and profusely branched. Plant height is 27.80–60 cm tall (50.08 ± 6.45 cm).	Erect, simple, or branched, 30–70 cm tall (60.67 ± 5.90 cm)
Leaves	Alternate, pinnatisect divided into three to nine lobes.	Alternate, two to three pinnate with very thin segments.
Inflorescence	Not involucrate.	Involucrate.
Flower	Terminal, solitary, star-shaped, 2–2.5 cm in diameter.	Terminal, solitary, 3.5–4.5 cm in diameter, surrounded by five involucral leaves forming involucres of bracts.
Sepal	Five petalloid sepals, lanceolate, light blue or white.	5–25 petaloid sepals, clawed, blue or white in color.
Petal	Eight reduced petals; each petal is formed of two bases enclosing nectar-pocket.	Eight petals, smaller than sepals, which are located at the base of the stamens.
Fruit	Follicles (capsule), green, dry, and brown at maturity, longer than wide. Formed from 3–10 carpals; each carpel ends with elongated style that persists after ripening of the fruit. Seeds are inserted one on the other in the fruit.	Follicles, green, dry, and brown in late summer, large and inflated, composed of several follicles welded to the top, containing seeds, with a horizontally spreading persistent style.
Seed	Small, obpyramidal form with a broad base and has a rough appearance; 3.16 ± 0.03 mm length and 1.77 ± 0.02 mm wide, black at maturity, with hard teguments and transversely ribbed.	Small, domed, 2.45 *±* 0.03 mm length and 1.73 *±* 0.01 mm wide, black at maturity and striated, transversely ribbed, with hard teguments.
Root	Well-developed yellow taproot.	Well-developed yellow taproot.

**Table 2:** Nutrient contents of *Nigella sativa* L. and *Nigella damascena* L. seeds (%).

Parameters	*Nigella sativa* L. Seeds	*Nigella Damascena* L. Seeds
Moisture	4.71 ± 0.09 ^a^	3.41 ± 0.03 ^b^
Crude fat	42.3 ± 0.67 ^a^	34.3 ± 0.35 ^b^
Crude protein	21.60 ± 0.35 ^b^	25.10 ± 0.74 ^a^
Total carbohydrates	27.23 ± 0.41 ^b^	32.47 ± 0.39 ^a^
Reducing sugars	6.90 ± 0.39 ^a^	5.62 ± 0.14 ^b^
Ash	4.16 ± 0.12 ^b^	4.72 ± 0.09 ^a^
Magnesium	0.01 ± 0.002 ^a^	0.01 ± 0.001 ^a^
Sodium	0.33 ± 0.03 ^b^	0.56 ± 0.02 ^a^

The results of *Nigella* species with different letters for each parameter indicate a statistically significant difference (Student’s *t*-test, *p* < 0.05, a > b).

**Table 3:** Phenolic content and antioxidant activity of NS (*Nigella sativa* L.) and ND (*Nigella damascena* L.) seeds.

Parameters	NS Seeds	ND Seeds	Reference Standards
**Phenolic contents**			
TEC (%)	20.64 ± 0.08 ^b^	29.94 ± 0.11 ^a^	-
TPC (mg GAE/100 g DW)	628.98 ± 4.38 ^b^	1841.46 ± 9.60 ^a^	-
TFC (mg QE/100 g DW)	59.87 ± 0.22 ^b^	138.45 ± 0.32 ^a^	-
Tannin (mg TAE/100 g DW)	44.54 ± 0.82 ^b^	69.58 ± 3.14 ^a^	-
**Antioxidant activity**			
Quenching of DPPH^•^ (IC_50_, mg/L)	617.52 ± 0.01 ^a^	318.84 ± 0.02 ^b^	75.65 ± 2.10 ^α^, 2.9 ± 0.02 ^β^, 70.6 ± 6 ^γ^
Quenching of ABTS^•+^ (IC_50_, mg/L)	82.92 ± 0.02 ^a^	44.15 ± 0.02 ^b^	2.465 ± 0.003 ^δ^, 1.244 ± 0.002 ^β^, 0.502 ± 0.001 ^ε^
Quenching of O2^•−^ (IC_50_, mg/L)	994.73 ± 0.02 ^a^	854.30 ± 0.01 ^b^	472 ± 10 ^δ^, 139 ± 6 ^β^, 84 ± 2GA ^ε^
Reducing power (A_0.5_, mg/L)	254.75 ± 0.03 ^a^	116.30 ± 0.01 ^b^	3.21 ± 0.001 ^β^, 1.79 ± 0.01 ^ε^

Abbreviations are as follows: TEC, total extractable compounds; TPC, total phenolic content; GAE, gallic acid equivalents; DW, dry weight plant material; TFC, total flavonoid content; QE, quercetin equivalents; TAE, tannic acid equivalents; α, butylated hydroxy anisole (BHA); β, quercetin; γ, ascorbic acid; δ, Trolox^®^; ε, gallic acid; IC_50_, the concentration of the extract necessary to scavenge 50% of radicals; A_0.5_, concentration of extract which gives an absorbance of 0.5.The results of *Nigella* species with different letters for each parameter indicate a statistically significant difference (Student’s *t*-test, *p* < 0.05, a > b).

**Table 4:** Phenolic acids and flavonoids identified and quantified from *Nigella sativa* L. and *Nigella damascena* L. seeds.

Phenolic Compound (mg/100 g Dry Weight)	*Nigella sativa* L.	*Nigella damascena* L.
**Phenolic acids**		
1. Gallic acid	1.69 ± 0.02 ^b^	17.86 ± 0.06 ^a^
2. Hydroxybenzoic acid	374.12 ± 0.37 ^a^	1010.71 ± 0.60 ^b^
3. Syringic acid	7.40 ± 0.05 ^a^	3.44 ± 0.06 ^b^
4. Vanillic acid	3.17 ± 0.01 ^b^	20.54 ± 0.08 ^a^
5. Caffeic acid	111.06 ± 0.21 ^a^	66.40 ± 0.11 ^b^
6. Coumaric acid	9.94 ± 0.09 ^a^	4.83 ± 0.06 ^b^
7. Cinnamic acid	1.55 ± 0.03 ^a^	1.52 ± 0.06 ^a^
**Flavonoids**		
8. Quercetin	0.15 ± 0.03 ^b^	1.42 ± 0.07 ^a^
9. Catechin	6.94 ± 0.05 ^b^	32.23 ± 0.06 ^a^

The results of *Nigella* species with different letters for each parameter indicate a statistically significant difference (Student’s *t*-test, *p* < 0.05, a > b).

**Table 5:** Biochemical parameters of mice plasma treated with and without *Nigella* extract (g/L).

Mice Group	Extract Dose (mg/kg)	Protein	Albumin	Glycemia	Urea	Cholesterol	Triglycerides
CTL	0	46.19 ± 0.05 ^a^	27.24 ± 0.01 ^a^	2.50 ± 0.03 ^a^	0.77 ± 0.03 ^a^	1.70 ± 0.01 ^a^	1.92 ± 0.04 ^a^
NS1	150	40.97 ± 0.01 ^b^	26.18 ± 0.04 ^b^	2.15 ± 0.06 ^b^	0.65 ± 0.02 ^b^	1.37 ± 0.02 ^bc^	1.81 ± 0.01 ^b^
NS2	500	40.59 ± 0.04 ^c^	25.27 ± 0.04 ^c^	2.00 ± 0.05 ^c^	0.56 ± 0.05 ^c^	1.32 ± 0.02 ^cd^	1.41 ± 0.03 ^c^
ND1	150	40.27 ± 0.03 ^d^	25.10 ± 0.06 ^d^	1.88 ± 0.02 ^d^	0.63 ± 0.06 ^bc^	1.39 ± 0.08 ^b^	1.80 ± 0.05 ^b^
ND2	500	40.04 ± 0.02 ^e^	24.78 ± 0.02 ^e^	1.58 ± 0.02 ^e^	0.57 ± 0.01 ^c^	1.30 ± 0.05 ^d^	1.45 ± 0.07 ^c^

Values are the means ± SD. Results in the same column with different letters are statistically different (Test-LSD, *p* < 0.05, a > b > c > d > e). Abbreviations are as follows: CTL, control group treated with the 0.9% saline solution; NS1, group of mice treated with 150 mg/kg body weight of *Nigella sativa* L. extract; NS2, group of mice treated with 500 mg/kg body weight of *Nigella sativa* L. extract; ND1, group of mice treated with 150 mg/kg body weight of *Nigella damascena* L. extract; ND2, group of mice treated with 500 mg/kg body weight of *Nigella damascena* L. extract.

**Table 6:** Antimicrobial activity of NS (*Nigella sativa* L.) and ND (*Nigella damascena* L.) extracts (expressed in mm).

Strains	NS Extract		ND Extract	
	5 mg/mL	195 mg/mL	5 mg/mL	195 mg/mL
**Gram-negative bacteria**				
*Escherichia coli*	n.a.	n.a.	n.a.	n.a.
*Pseudomonas aeruginosa*	n.a.	n.a.	n.a.	18 ± 0.50
**Gram-positive bacteria**				
Methicillin-resistant *Staphylococcus aureus*	n.a.	15 ± 0.30 ^a^	n.a.	14 ± 0.20 ^b^
Methicillin-sensitive *Staphylococcus aureus*	n.a.	14 ± 0.50 ^b^	n.a.	22 ± 0.50 ^a^
**Molds**				
*Aspergillus niger*	n.a.	n.a.	n.a.	n.a.
*Aspergillus flavus*	n.a.	16 ± 0.40 ^b^	n.a	20 ± 0.50 ^a^
*Mucor rammanianus*	n.a.	17 ± 0.40 ^a^	12 ± 0.20	14 ± 0.30 ^b^
*Aspergillus ochraceus*	n.a.	n.a.	n.a.	12 ± 0.50
*Aspergillus parasiticus*	n.a.	17 ± 0.30 ^b^	19 ± 0.40	22 ± 0.50 ^a^
**Yeast**				
*Candida albicans*	n.a.	13 ± 0.50	n.a.	n.a.

Here, n.a. indicates not active. Results with different letters in the same row are statistically different (Student’s *t*-test. *p* < 0.05; a > b).

## Data Availability

The data that support the findings of this study are available on request from the corresponding author.
